# Protein Sequence Alignment Analysis by Local Covariation: Coevolution Statistics Detect Benchmark Alignment Errors

**DOI:** 10.1371/journal.pone.0037645

**Published:** 2012-06-08

**Authors:** Russell J. Dickson, Gregory B. Gloor

**Affiliations:** Department of Biochemistry, The University of Western Ontario, London, Canada; University of Queensland, Australia

## Abstract

The use of sequence alignments to understand protein families is ubiquitous in molecular biology. High quality alignments are difficult to build and protein alignment remains one of the largest open problems in computational biology. Misalignments can lead to inferential errors about protein structure, folding, function, phylogeny, and residue importance. Identifying alignment errors is difficult because alignments are built and validated on the same primary criteria: sequence conservation. Local covariation identifies systematic misalignments and is independent of conservation. We demonstrate an alignment curation tool, LoCo, that integrates local covariation scores with the Jalview alignment editor. Using LoCo, we illustrate how local covariation is capable of identifying alignment errors due to the reduction of positional independence in the region of misalignment. We highlight three alignments from the benchmark database, BAliBASE 3, that contain regions of high local covariation, and investigate the causes to illustrate these types of scenarios. Two alignments contain sequential and structural shifts that cause elevated local covariation. Realignment of these misaligned segments reduces local covariation; these alternative alignments are supported with structural evidence. We also show that local covariation identifies active site residues in a validated alignment of paralogous structures. Loco is available at https://sourceforge.net/projects/locoprotein/files/

## Introduction

Multiple sequence alignments are critical for generating and testing hypotheses based on protein structure, function, and phylogeny. Protein alignments are built based on the assumption that each position (column) in the alignment is homologous [1]. With structural information, homology is typically validated by demonstrating that two residues occupy the same location in 3D space since structural homology implies sequential homology [2]. If only sequence information is available, positions are assigned based on the conservation of residue identity or properties, which is inherently less reliable than structural inference. The logic of interpreting sequence alignments is, therefore, circular: alignments are built, validated, and used based on a single criterion, conservation. A conservation-independent property of sequence alignments is a valuable adjunct to validate a sequence alignment.

Structure alignments are used to validate sequence alignments because they provide evidence independent of sequence; thus, benchmark datasets like BAliBASE include structural support [3], [4]. Unfortunately, structures are comparatively rare and cannot be used to validate all sequence alignments. In BAliBASE 3, there are many alignments that contain few structural seeds compared to the number of sequences. Furthermore, Kuziemko et al. noted that structurally supported alignments often do not score as highly as alignments that optimize the dynamic programming scoring function of sequence alignment algorithms, suggesting sequence alignment algorithms frequently reject the structurally valid alignment when such an alignment exists [2]. As sequence and structure grow more distant it becomes increasingly difficult to produce an alignment.

Multiple sequence alignment methods are typically benchmarked against high-quality datasets such as BAliBASE [3], [4]. In principle, BAliBASE alignments should represent the upper limit of quality that can be achieved using existing methods as they are both structure-aided and manually curated. Authors of sequence alignment algorithms strive to create alignments that are the most similar to the benchmark dataset. Benchmark datasets must be of the utmost quality to be reliable for assessing competing methods. However, Edgar demonstrated that inconsistencies and potential errors exist even in benchmark datasets like BAliBASE [5].

Another resource for hand-curated structure-based sequence alignments is the Conserved Domain Database (CDD) [6]. While CDD, was not originally designed to be a benchmark dataset like BAliBASE 3, its hand-curated structure alignments of are sufficient quality to be used as the benchmark dataset when analyzing structure alignment algorithms [7].

Alignments are also susceptible to errors for reasons independent of the circular logic of sequence alignment. Without careful manual curation, structure alignment algorithms are susceptible to shift error [7]. Shift errors are misalignments where the sequence has been shifted by 1 or more positions, though major secondary structural elements are still aligned. Since structure-based alignments are built by progressively aligning sequences to the seed structure alignment, shift errors can propagate systematically.

Progressive multiple sequence alignment strategies can also be prone to systematic propagation of errors as sequences are progressively added to a growing alignment. Iterative sequence alignment methods attempt to resolve this issue by employing a refinement step after the initial alignment is built. However, at present there is no method that reliably identifies shift errors. A disagreement between two theoretically valid alignment predictions is therefore very difficult to resolve; the current solution is to trust a benchmark dataset if available.

Covariation analysis is a statistical method used to understand coevolution in proteins [8]. Covariation can be understood intuitively as a measure of the reduction in uncertainty about one position given information about another. Covariation scores have minima when either both positions are absolutely conserved or when both positions are randomly assorting. A high covariation score implies that knowledge of one position provides information about the identity of the other.

Covariation statistics are used to indicate whether two residues are potentially coevolving [9]–[16]. Coevolving residues are thought to arise by a mechanism of constrained amino acid change [9], [17], [18]. Many covariation statistics predict contacting pairs with high accuracy [13]–[15], [19]. If this dependency between positions is due to some evolutionary process, like structural or functional constraints, then it is often defined as coevolution [20]. For clarity, coevolution is an evolutionary process, and covariation is the statistical non-independence used to identify it. When using covariation statistics to find coevolving pairs of positions a number of assumptions about the nature of the alignment are made; this includes the assumption that the protein family is properly aligned and all members are orthologous [15].

We previously demonstrated that with systematic sequence shifts (ie. synthetic misalignments), alignments show patterns of increased sequence-local covariation in the shifted segment [15]. We have extended the observations from [15] into an alignment curation tool called LoCo. LoCo is based on Jalview [21], [22] and provides a local covariation measure in real-time while curating an alignment. We use case studies to show how to apply LoCo to both the Conserved Domain Database [7] and BAliBASE 3 database [4] to identify sequence alignments that have regions of high local covariation. We provide examples of structurally validated realignments of the BAliBASE 3 benchmark dataset with both covariation and structural justification. Increased local covariation also identifies important functional residues in a structurally valid alignment from the BAliBASE 3 database. Finally, we demonstrate the method of investigating local covariation to determine if adjustments of the alignment is warranted.

## Results

### Illustrating How Covariation Identifies Sequence Shifts

**Figure 1:**
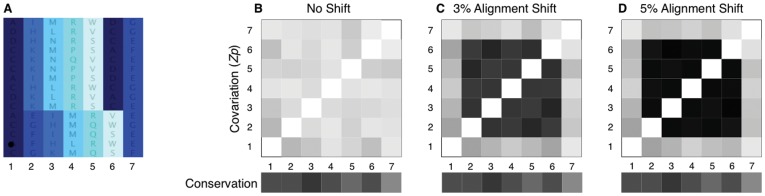
Local covariation identifies alignment shift errors. (**A**) A synthetic alignment was created for covariation analysis. Each of the 7 positions (columns) in the alignment contained a random assortment of 3 residues. A subset of the sequences (rows) in the alignment were then shifted for positions 2, 3, 4, 5, and 6 one position to the right. Position 1 and position 7 were not shifted. (**B**) A matrix of all pairwise covariation scores for the unshifted synthetic alignment where darker grey represents higher covariation calculated as in Materials and Methods. All positions randomly assort compared to one another; thus, panel **B** represents the background covariation for the synthetic block. Jalview conservation scores are also shown for each position. (**C**) Matrix of covariation scores where 3% of sequences (6 of 200) are shifted for positions 2–6. Covariation increases between all shifted positions, but does not increase between unshifted positions 1 and 7 and any of the unshifted positions. Conservation scores remain unchanged. (**D**) Matrix of covariation scores like panel **C**, except 5% of sequences are shifted. Covariation scores increase further between shifted positions, but unshifted positions show scores comparable to background as in panel **A**. Conservation scores remain unchanged.

The covariation statistic *Zp* (calculated as in Materials and Methods) is exquisitely sensitive to identifying residue non-independence in pairs of columns [15], [23]. To illustrate this effect, we created a 7-position synthetic alignment prepended to a 200 residue alignment of methionine aminopeptidase (Materials and Methods). Each column in the alignment is composed of a random assortment of 3 residues. Then, a small fraction of positions two through six were shifted 1 position to the right (Figure 1A). Positions 1 and 7 were not shifted and so were always randomly assorting relative to the other positions.

This alignment was loaded into the LoCo alignment viewer, which uses the existing Jalview codebase but replaces the Quality score with Local Covariation (Materials and Methods). Figure 1B (top) shows a heatmap of covariation scores when the statistic *Zp* [13] is applied to the synthetic block when no sequences are shifted (Materials and Methods). Darker shading represents higher conservation or covariation scores. Since all positions are randomly assorting and thus independent of one another, this heatmap represents the background covariation. The starting conservation scores, as calculated by Jalview, for the initial aligned positions are shown below. Figure 1B establishes a baseline for comparison; light grey implies a negligible covariation score.

The heatmap shown in Figure 1C shows all pairwise covariation scores when positions 2 through 6 contain 3% (6 of 200) shifted sequences. It is apparent that all pairwise covariation scores in the shifted region have increased compared to the baseline. Furthermore, the unshifted flanking positions, 1 and 7 (and all other unshifted positions in the MAP1 alignment), remain unchanged compared to the baseline shown in Figure 1B and have negligible covariation scores. Finally, Figure 1D shows that when 5% (10 of 200) of sequences are shifted, there is a marked increase in covariation scores in the misaligned region; also, there is no noticeable change in the amount of covariation between any unshifted positions. Finally, notice that conservation, which is the primary criterion on which alignments are built and evaluated, remains visibly unchanged in Figures 1B, 1C, and 1D.

The reason for increased local covariation in the shifted regions is the reduction of uncertainty between shifted positions [13], [15]. When two positions assort independently, as seen in Figure 1B, the knowledge of the residue present at a given position provides no information about any other position. However, when a block of sequence is shifted, positions are no longer independent, and positions in the same shifted block share predictive power. This illustration explains the observation in [15] that local covariation strongly correlates with systematic misalignments.

This simple illustration shows that local covariation easily identifies segments of alignments with these types of sequence shifts as described previously [15]. Previously [24], we used local covariation to identify a region that could assume either be alpha helical or beta stranded conformation within the orthologous phosphoglycerate kinase gene family. The remainder of this paper shows how local covariation can be used to identify other possible sources of high local covariation. As shown here, these can include putative systematic sequence misalignments and paralogous contamination of gene families.

### Identifying Alignments with High Local Covariation

**Figure 2:**
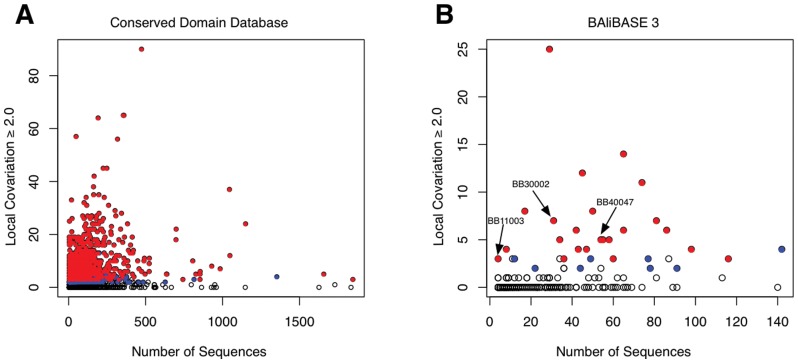
Alignments with high local covariation found in alignment databases. Each alignment in the Conserved Domain Database [6] and BAliBASE 3 [4] is represented by a single circle. Alignments are partitioned by the number of sequences and the number of regions of high local covariation. A region of high local covariation is defined as a local covariation peak greater than or equal to 2.0. Alignments with two adjacent regions of high local covariation are coloured blue. Regions that contain three or more contiguous regions of high local covariation are coloured red. (**A**) Analysis of all conserved domains (cd) in the Conserved Domain Database (CDD). (**B**) Analysis of all alignments in BAliBASE 3.

Local covariation is calculated as the mean covariation score over a window 6. If this mean score is greater than or equal to 2.0 then it is considered a high local covariation peak. Of the 6874 conserved domains (cd) analyzed in the CDD database (REF), 2189 had at least one peak at or above 2.0 (Figure 2A). We also analyzed the BAliBASE 3 benchmark database. Figure 2B shows that the majority of BAliBASE alignments do not have regions of increased local covariation. However, we found that 60 of the 217 alignments in BAliBASE 3 had at least one peak at or above the 2.0 local covariation threshold. Regions of high local covariation appear to be common in these alignment databases. We show below that these should be investigated manually to determine the root cause.

### Realigning a BAliBASE Multiple Sequence Alignment

In the BAliBASE 3 dataset, there were 37 alignments that contain contiguous blocks of high local covariation (filled dots). Of these, 30 had three or more contiguous high local covariation peaks, representing an extended range of high local covariation. We have chosen 3 alignments BB11003, BB30002, and BB40047 from 3 different categories of BAliBASE that demonstrate the characteristics of alignments with high local covariation. We illustrate how LoCo can be used to characterize the source of the high local covariation.

**Figure 3:**
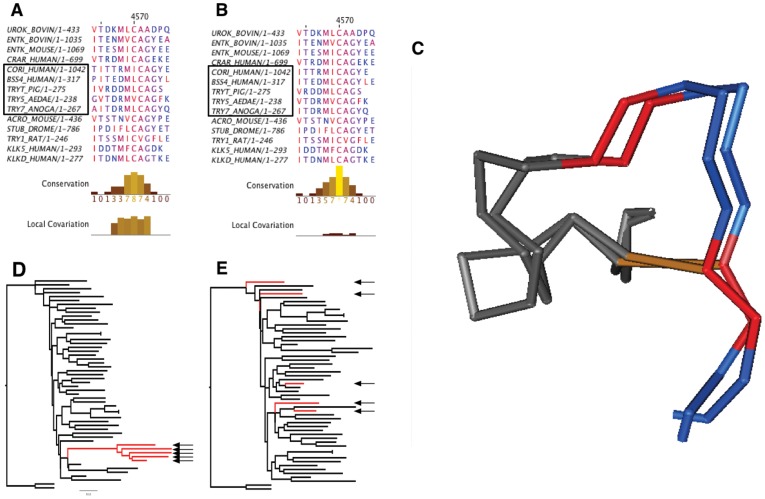
Realigning serine protease using LoCo. (**A**) Region of high local covariation and good conservation from alignment BB40047 from the BAliBASE 3 benchmarking dataset [4]. Five highlighted sequences do not show conservation of the disulphide bonded cysteine shown structurally in panel **C**. (**B**) Realignment of region from panel **A** using local covariation as a guide. (**C**) Structural validation of the alignment from panel **B** built in Cn3D [39]. Positions homologous to those shown in panels **A** and **B** are coloured by identity; the conserved disulphide bond is highlighted in orange. (**D**) Neighbour joining tree of high local covariation segment shown in panel **A**. Potentially misaligned sequences (indicated by arrows) cluster in a clade joined to the remainder by a long branch. (**E**) Neighbour joining tree based on realigned segment in **B** shows realigned sequences no longer cluster together as expected by the phylogenetic relationship of the organisms.

We identified a contiguous segment of high local covariation in the BB40047 alignment of BAliBASE 3. BB40047 is built upon the alignment of two structures containing a disulphide bond shown in Figure 3C. Figure 3A, a screenshot from the LoCo tool, shows the sequence alignment corresponding to the coloured region of the structure in Figure 3C. The region highlighted is the only block showing increased local covariation in this alignment. The BAliBASE alignment does not show conservation of the disulphide bonded cysteine; the presence of a cysteine is necessary to maintain the disulphide bond.

Although there is no structural information for the highlighted sequences, we can infer that the adjacent cysteine should be aligned to the disulphide bonded position because the existing alignment would place the cysteine in a conformation unable to form a disulphide bond. Figure 3D shows that the highlighted sequences group together when the region of high local covariation is clustered using the built-in Jalview function for neighbour joining tree by percent identity. Using the procedure outlined in the Methods section, the region can be adjusted as shown in Figure 3B. The adjusted alignment shows perfect conservation of the cysteine that is absolutely necessary for maintaining the disulphide bond shown structurally in Figure 3C. The adjusted alignment also shows a marked decrease in local covariation. After the sequences have been adjusted, they no longer cluster together (Figure 3E). Instead, clustering is more similar to that expected by the organism relationships.

Thangudu et al. noted that imperfect conservation of disulphide bonds in alignments is frequently caused by structure or sequence alignment errors [25]. The decrease in local covariation comparing the original BAliBASE (Figure 3A) with the realigned (Figure 3B) and the absolute conservation of the disulphide bond illustrates how LoCo can be used for identifying potentially troublesome sites.

### Realigning a BAliBASE Structure Alignment

**Figure 4:**
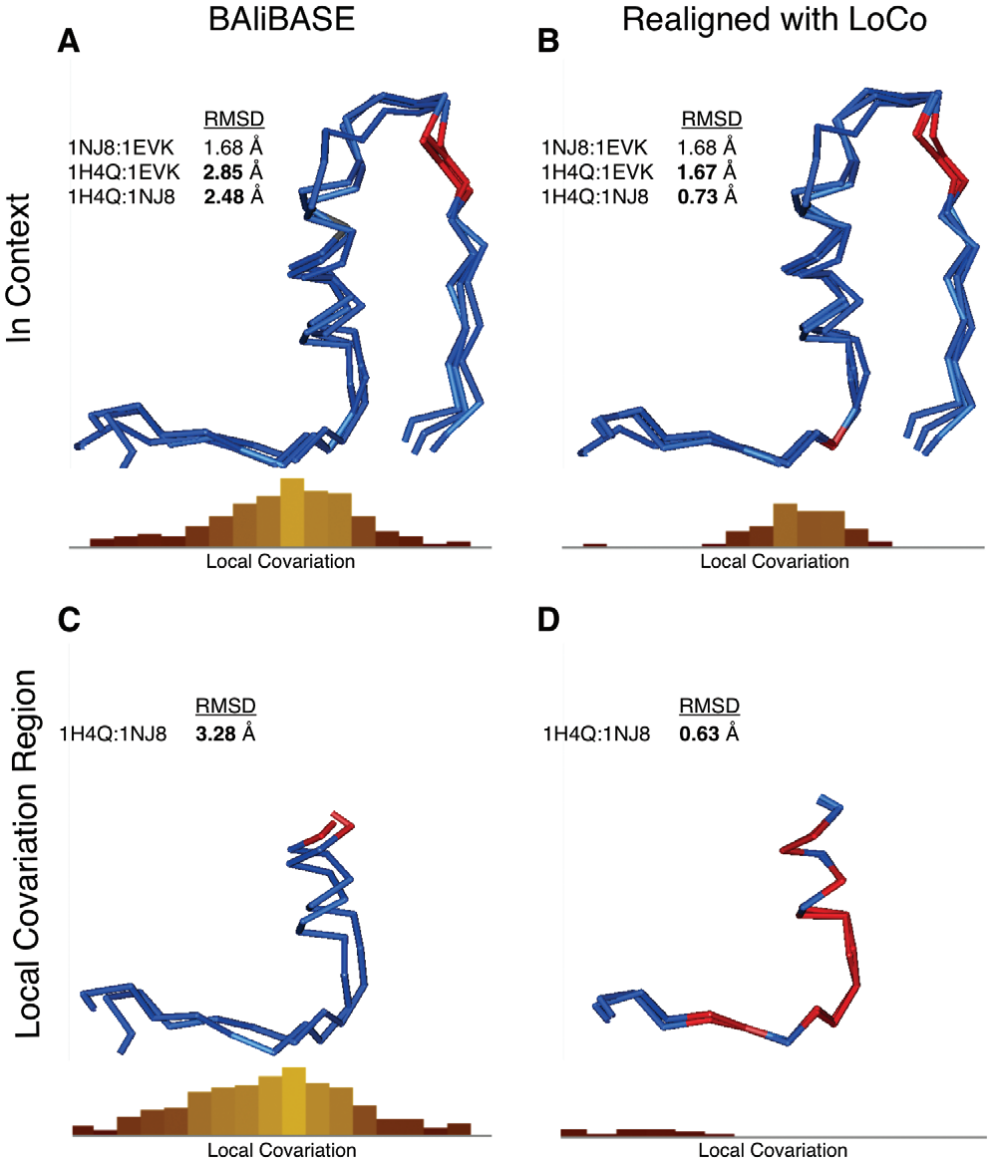
Local covariation identifies structural alignment error in BAliBASE 3 alignment of tRNA Synthetases (BB30002). Each panel shows a structure alignment built with Cn3D [39] with the corresponding local covariation histogram from LoCo below. (**A**) Structure alignment of the tRNA synthetase subfamilies from BAliBASE 3. Structures are coloured by fit and the maximum local covariation value (2.8) implies a misalignment exists. (**B**) Realignment of misaligned structure from panel **A** reduces local covariation (maximum peak 1.8). Both panels **A** and **B** look very similar which explains why misalignment was missed during BAliBASE manual curation process. (**C**) Structure alignment of *only* the misaligned region of Prolyl tRNA Synthetase subfamily from panel **A**. Structures are coloured by identity such that conserved residues are red. Local covariation maximum is 3.0. (**D**) Realignment of panel **C** to minimize local covariation. Minimizing local covariation produces marked improvement in both the structure alignment quality and sequence conservation.

Some structure alignments generated by unsupervised algorithms suffer from shift error [7] where the location of secondary structures are aligned correctly, but pairwise alignment of residues is offset relative to the periodicity of the secondary structural element. Such alignments can be difficult to identify visually or by root-mean-square deviation (RMSD) because the unshifted alignment preceding and following the misalignment can create the appearance of correct alignment; as well, the misalignment can be obscured by other structures. We demonstrate this type of an erroneous structure alignment in Figure 4.

Local covariation analysis of BAliBASE 3 identified a region of interest in the alignment BB30002 (Figure 2). BB30002 is particularly difficult to analyze because it is an alignment of several paralogous tRNA synthetases. In intra-molecular coevolution analyses, paralogous sequences are seen as contamination and can lead to false-positve conclusions since their presence violates the implicit assumptions of coevolutionary analyses [26]. The BAliBASE alignment of structures representing prolyl- and threonyl-tRNA synthetases are shown in Figure 4A. Visual inspection of the structure alignment suggests the region is well-aligned. However, Figure 4B shows an alternative alignment with lower local covariation. The structure alignments shown in Figure 4A and Figure 4B appear to be of equivalent quality when visually inspected. However, the realigned structures in Figure 4B show an improvement to the RMSD scores. The RMSD of the orthologous structures, 1H4Q and 1NJ8, improves from 2.44 Å to 0.73 Å. The RMSD of the paralogous structures, 1H4Q and 1EVK, improves from 2.85 Å to 1.67 Å. Thus when aligning divergent structures, misalignments may be undetectable by visual inspection.

In Figure 4C and D, we analyze only the orthologous sub-family to clarify the structure misalignment visually. Figure 4C shows the alignment of the high local covariation region of only the prolyl-tRNA synthetase subfamily of BB40002. This region shows poor structural conservation and residue identity. When the shift error is resolved using LoCo as a guide, the quality of the alignment is markedly improved (Figure 4D). The alignment shows improved sequence conservation and a much lower RMSD, from 3.28 Å to 0.63 Å. The local covariation present in Figure 4C is no longer present in Figure 4D.

The BB30 category of alignments are designed to test the ability to properly align multiple subfamilies into a single subalignment. Aligning paralogous sequences is particularly challenging because of increased sequence divergence and different functional constraints. Functional divergence can result in increased substitution rates (type I divergence) [27]. Divergence can also occur without a change in substitution rate in the form of differing residue properties allowed at a given position (type II divergence) [28], [29]. These types of divergence can make it difficult to determine the alignment between paralogous proteins from sequence alone. However, the misalignment presented in BB30002 is within a subfamily and is between two structures. The discovery of a structural misalignment between two similar sequences from the same subfamily in a hand-curated alignment demonstrates the importance of independent validation of sequence and structure alignments.

### Local Covariation Identifies Active Site Residues

**Figure 5:**
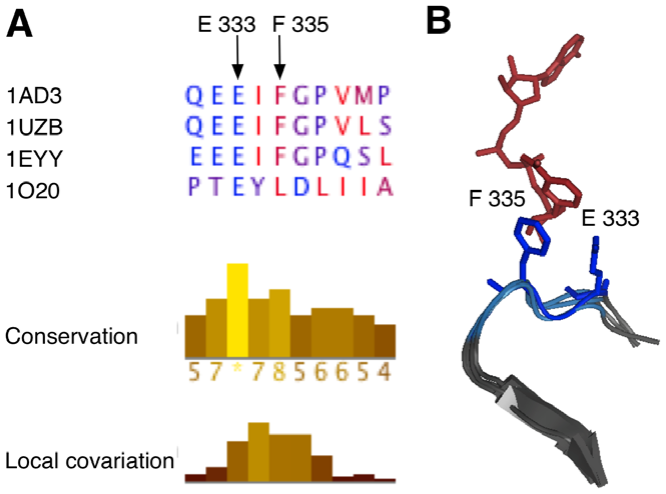
Local covariation identifies active site residues. (**A**) Screenshot from the LoCo tool showing the region of high local covariation from BAliBASE 3 alignment BB11003. BB11003 is an alignment of four paralogous oxireductases with similar structure. The local covariation peaks highlight four positions in the sequence alignment which are coloured in blue in panel **B**. Two active site residues from structure 1AD3, E333 and F335, are emphasized in the sequence alignment. (**B**) Structure alignment of residues shown in panel **A** made in PyMOL [40]. The region of high local covariation is highlighted in blue; structure 1AD3 is emphasized with dark blue. The NAD cofactor from structure 1AD3 is drawn in red. Important binding residues E333 and F335 from 1AD3 are rendered in sticks representation.

Not all regions of high local covariation in BAliBASE are explained by potential misalignments; in fact, some segments with high local covariation are structurally valid. It is thus crucial to examine regions of high local covariation to determine the root cause. As outlined in this section, local covariation can identify segments of interest that covary because of another mechanism. In our analysis of BAliBASE 3, we identified BB11003 as an alignment with a region of high local covariation(Figure 5). Two structures, 1AD3 and 1EYY, are of aldehyde dehydrogenase; the other structures are of carboxylate dehydrogenase (1UZB), and 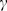-glutamyl phosphate reductase (1O20). We investigated this alignment for an explanation of the high local covariation. The sequence alignment in the region of high local covariation (Figure 5A) is supported by the structure alignment in the same region (Figure 5B). Thus, we concluded that shift error did not explain the high local covariation.

As noted in the previous section, protein families undergo functional divergence after gene duplication leading to paralogous alignments have specific characteristics. Functional divergence that occurs at a only a clustered subset of positions will cause an increase in local covariation. Thus, the presence of paralogues that have undergone type II divergence [28], [29] may create a false-positive detection of misalignment. However, we show that the detection of type II divergence in important functional regions may prove useful for identifying binding sites or understanding divergence in paralogous families.

Structure 1AD3 included the coenzyme NAD, which is critical for enzyme function. Figure 4B shows that the region of high local covariation (blue) is oriented towards NAD (red). The region of high local covariation spans four residues: E333, I334, F335, and G336. E333 is absolutely conserved and therefore cannot contribute to any covariation score. The other three residues, I334, F335, and G336, vary in the sequence of 1O20 but not in the backbone structure. Because NAD is critical for catalysis [30], we hypothesized that the contacts made by E333 and F335 could be important for function [31].

The human homologues for positions E333 and F335, E399 and F401 respectively, have been found to be important for function. The human E399 binds the NAD ribose; mutations to the position significantly affect the catalytic rate [32]. F335 orients NAD through an aromatic stacking interaction Figure [31]. Thus, local covariation identified important functional residues from a paralogous protein family. This example illustrates that not all regions of high local covariation are caused by misalignments. Thus, it is important to visually inspect regions of high local covariation to elucidate the cause.

## Discussion

Protein family misalignments can cause errors in downstream analyses — unimportant positions may be falsely identified as conserved or coevolving and critical conserved positions can be overlooked. Systematic misalignments can reduce the bootstrap values of phylogenetic trees or reinforce incorrect trees [33]. Thus, it is critical that alignments be validated by a criterion independent of the assumptions used to build them.

Selecting which alignment is most likely correct can be a source of debate because there is no high-throughput biochemical method to prove the validity of an alignment. Some investigators prefer to believe the internally consistent output of an established alignment algorithm over an alternative alignment with some biological justification. Here we provide a tool to identify regions in an alignment that should be investigated. Automating alignment using local covariation as a parameter is difficult because increased local covariation is not tautologically equivalent to misalignment, as shown by the example of correctly aligned paralogs in Figure 5. However, as a guide for curation of protein alignments, the tool is extremely effective at identifying regions of potential misalignment [15], [24], [26], [34].

We provide strong structural evidence of the validity of our alternative alignments over the BAliBASE alignments in the form of cysteine conservation at a disulphide bond (Figure 3) and significantly improved RMSD of a structure alignment (Figure 4). As noted by Kuziemko et al., the alignment supported by structural evidence may receive a lower score than an alignment which simply optimizes the sequence alignment algorithm's scoring function [2]. This observation suggests that we should be skeptical of alignments that are validated only by an alignment scoring function. Furthermore, the existence of potential misalignments in the most widely used, hand-curated benchmark dataset implies that such misalignments may be common in high-throughput datasets of lower quality.

Large datasets are known to have many systematic misalignments caused by incorrect sequential or structural inference because of the limitations of current alignment methods [5], [7]. Many alternative alignments may seem equally valid because there are no methods to prove the correct alignment aside from solving the structures for all proteins in the alignment. Thus, identification of serious errors with significant contradictory structural evidence is a method for demonstrating an alignment is incorrect. Such structurally corroborated misalignments are rare, especially in curated datasets. Nevertheless, the misalignments we identified in Figure 3 and Figure 4 provide such structural evidence.

It is interesting to contrast this assessment of BAliBASE 3 with a previous analysis of BAliBASE by Edgar [5]. Both studies investigate the quality of alignment benchmarks using criteria independent of sequence conservation. The different criteria for evaluating BAliBASE highlighted different sets of BAliBASE alignments for discussion. Edgar used domain homology and secondary structure annotations to assess alignment quality; he argues correctly that alignments of sequences with conflicting annotations are less reliable for benchmarking. In this manuscript, we identify structurally supported shift errors in the same dataset and, by extension, other similar datasets. These two studies form complementary assessments of the BAliBASE benchmark set.

The exploration of the BAliBASE BB30 subfamilies dataset, as in Figure 4, draws attention to the concept of homology and sequence alignment. Alignments designed to search for coevolving positions in a protein should ideally be orthologous, comprising sequences related by linear descent. However, sequences can also be homologous (similar by common evolutionary history) because of paralogy (related through a gene duplication event). Paralogous positions may be under different functional constraints [27]–[29]; an example would be the tRNA synthetases shown in Figure 4A and B. While both subfamilies are tRNA synthetases, they catalyze a reaction with different tRNAs and different amino acids. Although more exploration is needed, the inclusion of paralogous sequences could potentially increase local covariation to a lesser extent than misalignments. The presence of paralogous sequences may explain the occurrence of covariation within binding sites.

Identifying functional residues is an important open problem. The degree of conservation of a position is typically used to indicate its potential importance in such analyses. However, when paralogous families are included and conservation is lost, local covariation could also be used to search for non-conserved, functionally important residues. We provide an example of local covariation in a functional region in our analysis of the alignment BB11003. Investigating the region of local covariation revealed two important functional residues in an alignment of 4 sequences. Residues E333 and F335 both make important contacts to NAD in the coenzyme binding site (Figure 5).

Local covariation previously identified an interesting structural region in phosphoglycerate kinase [24]. In this example, a linker region contained either a sheet or a helix to serve the same structural purpose. Technically, the region was not shifted because there was no alternative alignment; there was simply no structurally meaningful alignment between the two sequence subsets. These examples illustrate that it is critical that alignments be visually inspected regardless of the method used to generate them.

An interesting illustration of the importance of manual alignment curation is provided by Kawrykow et al. through their work on the sequence alignment game Phylo [35]. Phylo uses the concept of crowdsourcing to improve sequence alignments by having human players inspect and correct them. It is important to note that Kawrykow et al. found that untrained game players were able to outperform the top performing automated solutions. This observation reinforces the importance of visually inspecting alignments after they are built by an automated solution; LoCo provides an interface to guide and expedite the investigation.

Increased local covariation should not be confused with patch covariation, where two short contiguous segments of sequence coevolve with one another [19]. Increased local covariation is only concerned with covariation that occurs within a short segment of an alignment, not between segments. As we noted previously, it is possible to use covariation statistics like 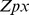 and 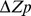 to find true coevolving pairs that are distant in sequence even in regions of misalignment [15].

We have made the tool used in this manuscript, LoCo, available online. LoCo can be used effectively on large datasets. Performance can become a concern when analyzing alignments with many ungapped positions because of the covariation calculations. However, because the covariation algorithms are implemented in C and optimized, we have successfully analyzed very large concatenated protein datasets with thousands of sequences. We have run LoCo successfully on concatenated alignments over 2500 ungapped positions long, though at this size the covariation module requires approximately 1 gigabyte of memory and 1 minute of CPU time to update the local covariation score. Alignments this size can be analyzed because of the extensive optimizations made to the covariation calculation software. LoCo and its antecedents have been an important part of building high quality protein alignments for several recent manuscripts [15], [24], [26], [34]. Using LoCo, we have seen marked improvement in our sequence alignment quality, confidence, and downstream analyses.

Analyses of alignments which contain errors are inherently unreliable. LoCo provides an intuitive and rapid platform to identify and correct alignment errors. We recommend that new alignments be analyzed with local covariation and visually inspected before any conclusions are drawn from them.

## Materials and Methods

### Demonstrating Local Covariation Rationale

We created a 7-position synthetic alignment to demonstrate the effectiveness of local covariation for finding misalignments (Figure 1). Each column in the misalignment contained a randomly assorted subset of 3 residues that was mutually exclusive with adjacent columns; this alignment was called ‘No Shift’. The ‘3% Alignment Shift’ and ‘5% Alignment Shift’ aligments were created by randomly shifting a subset of sequences one position to the right, 6 of 200 and 10 of 200, respectively. Figure 1A shows the shift of positions 2–6 diagrammatically. Positions and 1 and 7, which flank the misaligned region, remain unshifted.

The synthetic alignments were inserted at the N-terminus of a structure-guided and manually-curated alignment of methionine aminopeptidase. We subsequently analyzed the synthetic alignment using the covariation statistic *Zp* [13], [15] Conservation scores were calculated using Jalview [22].

### Algorithm Overview

LoCo calculates the average covariation between positions in a protein alignment using the *Zp/MIp* statistic [13] using a compiled program written in C. The algorithm for calculating *Zp* is optimized for memory use and speed. *Zp* is based on mutual information, a statistic that is calculated based on the relative counts and pairwise counts of each individual alignment position.

*MIp* is defined as:
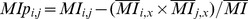
(1)where 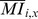 is the mean Mutual Information of position 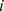 with all other positions and 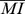 is the overall mean Mutual Information. *MIp* is normalized and referred to as *Zp*:
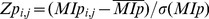
(2)where again 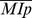 is the mean *MIp* and 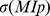 is its standard deviation. The convention of referring to normalized *MIp* as *Zp* was introduced in [15].

Because there are 20 amino acids, there are 20 potential entries in the count matrix; each pairwise count represents two positions so there are 400 potential entries for each pairwise count. However, because the majority of positions demonstrate some degree of conservation, most entries in the count and pairwise count matrices will be zero. This fact is exploited by the LoCo algorithm — a reusable linear array is used to initialize a dynamically allocated linked list which stores the pairwise count for each pair of positions for significant memory savings.

Local covariation is calculated by taking the average *Zp* score between all pairs of positions over a window of six; this is done in a Perl script upon completion of the C program.

The programs used to calculate covariation statistics can be used independently of the Jalview GUI. These programs are accessed using the Perl script MIp.pl; they take a fasta-formatted alignment and, optionally, a pdb-formatted structure as input and return a summary file of covariation statistics (and inter-residue distances if the pdb file is provided). The MIp software can be automated to screen large alignment datasets.

### The LoCo Alignment Curation Tool

The alignment editing software is a modified version of Jalview [22]. Because covariation statistics can be time-consuming to calculate, the major calculations are computed using an optimized algorithm implemented in the C programming language. The default Jalview sequence alignment window displays protein sequences above three indicators of alignment quality — Conservation, Quality score and Consensus. Because quality scores are based on conservation, in LoCo we have replaced Quality with Local Covariation. High local covariation indicates a high likelihood of systematic misalignment in that region, regardless of conservation score.

### The LoCo Alignment Curation Procedure

We have developed a simple procedure to correct potential systematic misalignments using LoCo: 1) Identify potential misalignments (Figure 3A), 2) cluster using neighbour joining by percent identity (Figure 3D), 3) test alternate alignments (Figure 3B).

Potentially misaligned regions can be identified by examining the “Local Covariation” bar at the bottom of the alignment window. In [15], we noted that a local covariation score above 2.5 was worth investigating; however, we have found that cutoff to be conservative. Covariation scores are affected by the number of sequences in the alignment and by their similarity, so it is possible to find misalignments in small alignments (approximately 10 sequences) with much lower local covariation scores. Alignments with fewer sequences have narrower distributions of covariation. We recommend investigating any position where the local covariation score 1) appears to be above the ‘background’ for the alignment, 2) is increased for several adjacent positions, or 3) is above 2.0 (coloured yellow in the histogram).

Clustering is done by highlighting the potentially misaligned positions and selecting “Neighbour Joining Using % Identity” from the Calculate menu. Regions of systematic misalignment will cluster separately from correctly aligned sequences. Sequences can be placed in the same order as the tree by using the Sort command in the Calculate menu.

Finally, alternate alignments can be tested by highlighting the region of misalignment and dragging the misaligned sequence into position by holding control while left-clicking and dragging the mouse. The local covariation score will change as you edit the alignment.

### Automated Search of CDD and BAliBASE

We collected alignments from the Conserved Domain Database [6] from

ftp://ftp.ncbi.nih.gov/pub/mmdb/cdd/

We collected all sequences from the ftp distribution of BAliBASE 3 [4] from

ftp://ftp-igbmc.u-strasbg.fr/pub/BAliBASE3/

The BAliBASE alignments were converted to fasta format by readseq [36]. A simple Perl-based pipeline was used to automate the use of the MIp.c and MIp.pl programs used to calculate covariation in the LoCo alignment curation tool. We counted the number of local covariation peaks at or above the 2.0 threshold considered worth investigating. The number of peaks above 2.0 were plotted in R [37]; contiguous blocks were coloured as they represented an extended region of high local covariation.

### Structure Validation

Structures were collected from the RCSB Protein Data Bank [38]. Structure alignments for Figure 3 and Figure 4 were made using Cn3D [39]. The Cn3D alignments are coloured by identity such that conserved positions are coloured red and non-conserved positions are coloured blue. RMSD for structure alignments was calculated using PyMOL [40]. The structure alignment for Figure 5 was created using PyMOL [40]. The entire structure alignment was rendered using the ‘cartoon’ renderer. Important residues and the NAD cofactor are emphasized through stick rendering on top of the original alignment. NAD is coloured red. The region of high local covariation is coloured blue.
